# Eicosapentaenoic and docosahexaenoic acid supplementation and inflammatory gene expression in the duodenum of obese patients with type 2 diabetes

**DOI:** 10.1186/1475-2891-12-98

**Published:** 2013-07-15

**Authors:** Marie-Ève Labonté, Patrick Couture, André J Tremblay, Jean-Charles Hogue, Valéry Lemelin, Benoît Lamarche

**Affiliations:** 1 Institute of Nutrition and Functional Foods, Laval University, 2440 boul. Hochelaga, Québec (Qc) G1V 0A6, Canada; 2 Department of Gastroenterology, Laval University Hospital Research Center, 2705, boul. Laurier, Québec (Qc) G1V 4G2, Canada

**Keywords:** Eicosapentaenoic acid (EPA), Docosahexaenoic acid (DHA), Signal transducer and activator of transcription 3 (STAT3), Inflammatory gene expression, n-3 supplementation, Placebo-controlled, Duodenum, Type 2 diabetes

## Abstract

**Background:**

The extent to which long-chain omega-3 polyunsaturated fatty acids (LCn-3PUFA) from fish oil such as eicosapentaenoic acid (EPA) and docosahexaenoic acid (DHA) exert their anti-inflammatory effects by down-regulating intestinal inflammation in humans is unknown. We investigated the impact of LCn-3PUFA supplementation on inflammatory gene expression in the duodenum of obese patients with type 2 diabetes.

**Findings:**

This placebo-controlled randomized crossover study included 12 men with type 2 diabetes. After a 4-week run-in period, patients received in a random sequence 5 g/d of fish oil (providing 3 g of EPA + DHA) and a placebo (corn and soybean oil) for 8 weeks each. The two treatment phases were separated by a 12-week washout period. Gene expression was assessed by real-time polymerase chain reaction in duodenal biopsy samples obtained in the fasted state at the end of each treatment phase. Intestinal mRNA expression levels of interleukin (IL)-6 and tumor-necrosis factor (TNF)-α were hardly detectable after either treatment (<100 copies/10^5^ copies of the reference gene ATP5o). Intestinal mRNA expression of IL-18 and of the transcription factor signal transducer and activator of transcription 3 (STAT3) was higher (>5000 copies/10^5^ copies ATP5o) but still relatively low. EPA + DHA supplementation had no impact on any of these levels (all *P* ≥ 0.73).

**Conclusions:**

These data suggest that duodenal cells gene expression of pro-inflammatory cytokines is low in patients with type 2 diabetes and not affected by EPA + DHA supplementation. Further studies are warranted to determine if inflammatory gene expression in other tissues surrounding the intestine is modulated by EPA + DHA supplementation.

**Trial registration:**

ClinicalTrials.gov ID: NCT01449773

## Findings

### Introduction

It is widely recognized that obesity, metabolic syndrome, and type 2 diabetes are associated with low-grade chronic inflammation [1], attributed in part to an expanded adipose tissue mass infiltrated with macrophages that secrete pro-inflammatory cytokines [2,3]. It has been suggested that diet-induced inflammation in the small intestine is also linked to obesity and insulin resistance [4]. Long-chain omega-3 polyunsaturated fatty acids (LCn-3PUFA) such as eicosapentaenoic acid (EPA, C20:5) and docosahexaenoic acid (DHA, C22:6) have been shown to have anti-inflammatory effects by down-regulating inflammatory gene expression in adipocytes and mononuclear cells [5-7]. Intervention studies evaluating the extent to which EPA and DHA may exert their anti-inflammatory effects by down-regulating intestinal inflammation are lacking. The aim of the present study was to investigate the impact of EPA and DHA supplementation on the expression of inflammatory genes in the duodenum of obese men with type 2 diabetes, who are likely to have intestinal inflammation [4] and therefore to respond favorably to LCn-3PUFA supplementation [8]. We hypothesized that EPA and DHA supplementation reduces pro-inflammatory gene expression in the duodenum of obese men with type 2 diabetes.

## Methods

### Participants

Twelve adult men with type 2 diabetes were recruited via the Lipid Clinic of the Laval University Hospital Research Center, Québec, QC, Canada, between April 2008 and March 2009. Diagnosis of type 2 diabetes was based on criteria issued by the American Diabetes Association [9]. Inclusion criteria were: age between 18 and 55 y, plasma triglyceride (TG) levels above the 50th percentile for age [10], non-smoker, body mass index (BMI) between 25.0 and 40.0 kg/m^2^, stable body weight over the last 6 mo, hemoglobin A1c (HbA1c) between 6.5 and 8.5%, baseline fasting plasma glucose < 15.0 mmol/L and patients with *de novo* type 2 diabetes not taking oral hypoglycemic agents or patients having received stable doses of metformin for at least 3 mo before randomization. Exclusion criteria were: genetic dyslipidemias, patients with secondary form of diabetes or acute metabolic diabetic complications, subjects having cardiovascular diseases or taking medication known to affect lipoprotein metabolism (e.g. lipid lowering agents). All participants signed an informed consent document approved by the Ethics Board of the Laval University Hospital Research Center.

### Study design

The study was undertaken according to a double-blind randomized crossover design with two balanced [1:1] treatments of 8 weeks each. All subjects received, in random order: 5 g/d (5 × 1 g capsules) of fish oil providing 3 g/d of EPA (64%) and DHA (36%) or a control supplementation (5 × 1 g capsules/d of a 50/50 blend of corn and soybean oil). Treatments were separated by a 12-week washout. During a 4-week run-in stabilization period that preceded the treatments, participants were advised to consume a low fat diet following the recommendations of the National Cholesterol Education Program - Adult Treatment Panel III [11]. Dietary intake of marine-derived LCn-3PUFA was limited by prohibiting the consumption of fish during the entire experimental period, including the washout period. Alcohol consumption, vitamin supplements and natural health products were also strictly forbidden during the entire experimental period.

Participants’ baseline intake of marine-derived LCn-3PUFA was assessed using a validated interviewer-administered food frequency questionnaire [12]. Compliance to supplementation was assessed by counting the number of capsules returned to the research staff over the course of the experimentation.

### Characterization of plasma lipids

Plasma TG concentrations were determined by enzymatic methods at the end of each supplementation period using the Technicon RA-1000 analyzer (Technicon Instruments Corporation, Tarrytown, NY). Plasma phospholipids fatty acid levels were determined as previously described [13].

### Duodenal biopsies

Four biopsy samples (3 × 3 mm) were obtained at the end of each phase by gastro-duodenoscopy from the second portion of the duodenum using multiple sample single-use biopsy forceps and were immediately flash-frozen in liquid nitrogen and stored at −80°C, as previously described [14].

### Total RNA extraction

Biopsy samples were homogenized in 1 ml of Qiazol. Ribonucleic acid (RNA) was then extracted using an RNeasy mini-kit (Qiagen). Tissue samples were also treated with an RNase-free DNase set to eliminate any contaminant deoxyribonucleic acid (DNA). Total RNA was then eluted into 100 μl RNase-free H_2_O and stored at −80°C.

### RNA quantification and quantitative real-time PCR

Details on RNA quantification and quantitative real-time PCR are provided in Additional file 1. Briefly, RNA quality was assessed with a 2100 Bioanalyzer (Agilent Technologies, Inc.) as previously described [15]. Messenger RNA (mRNA) expression data were normalized using the second derivative and double correction method [16] and are expressed as the number of copies/10^5^ copies of the reference gene ATP synthase O subunit (ATP5o). The standard curve was established by using known amounts of purified polymerase chain reaction products and the Light Cycler 480 version 1.5 software provided by the manufacturer (Roche Inc).

### Statistical analyses

Nonparametric Wilcoxon matched-pairs signed-rank tests were used to compare the impact of LCn-3PUFA and placebo on inflammatory gene mRNA expression in duodenal tissues. Mixed models with proper interaction terms have shown no evidence of a carry-over effect of the LCn-3PUFA supplementation (not shown). Associations among key outcomes were assessed using Spearman correlation coefficients. Differences were considered significant at *P* < 0.05. Analyses were performed using SAS (version 10.1; SAS Institute, Cary, NC).

## Results

All 12 recruited participants completed the study and were included in the analyses. Table 1 shows their characteristics. Body weight remained stable throughout the study. More than 80% of the capsules were taken by participants. No side effects were reported. Plasma phospholipids LCn-3PUFA levels were higher while plasma phospholipids levels of dihomo-γ-linoleic acid and arachidonic acid were lower after EPA + DHA supplementation than after placebo (all *P* ≤ 0.001). This again reflected good compliance. There was no between-treatment difference in alpha-linolenic acid and linoleic acid levels (both *P* ≥ 0.09, Table 2). Mean plasma TG concentrations tended to be lower after EPA + DHA supplementation than after placebo (2.30 ± 1.09 vs. 2.50 ± 1.22 mmol/L; *P* = 0.08).

**Table 1:** **Characteristics of the study participants at screening (*n*** **= 12 men with type 2 diabetes)**

	**Mean ± SD**
Age (y)	54.1 ± 7.2
BMI (kg/m^2^)	33.7 ± 6.0
LDL-C (mmol/L)	2.9 ± 0.5
HDL-C (mmol/L)	1.0± 0.2
TG (mmol/L)	3.1 ± 2.0
HbA1c (%)	7.0 ± 0.8
EPA + DHA intake (g/d)	0.23 ± 0.20

Abbreviations: *BMI* body mass index, *DHA* docosahexaenoic acid, *EPA* eicosapentaenoic acid, *HbA1c* hemoglobin A1c, *HDL*-*C* high density lipoprotein cholesterol, *LDL-C* low-density lipoprotein cholesterol, *TG* triglycerides.

**Table 2:** Plasma phospholipids fatty acid levels after EPA + DHA and placebo supplementation in 12 men with type 2 diabetes

	**Placebo**	**EPA + DHA**	**Difference**	***P***
	**Fatty acid levels (%)**			
LA	19.9 (3.6)	19.3 (2.9)	−0.6	0.09
ALA	0.21 (0.09)	0.19 (0.11)	−0.02	0.85
DGLA	3.82 (1.57)	2.87 (0.65)	−0.95	0.001
AA	9.40 (2.05)	7.97 (1.37)	−1.43	0.0005
EPA	1.00 (0.93)	3.53 (1.65)	2.53	0.0005
DPA	1.03 (0.34)	1.25 (0.26)	0.22	0.0005
DHA	2.96 (1.15)	4.43 (1.26)	1.47	0.0005

Data are expressed as median and interquartile range in parentheses. *P* values were calculated using Wilcoxon matched-pairs signed-rank tests. Abbreviations: *AA*, arachidonic acid; *ALA*, alpha-linolenic acid; *DGLA*, dihomo-γ-linoleic acid; *DHA*, docosahexaenoic acid; *DPA*, docosapentaenoic acid; *EPA*, eicosapentaenoic acid; *LA*, linoleic acid; *PUFA*, polyunsaturated fatty acids.

Table 3 presents the duodenal mRNA expression of inflammatory genes measured after placebo and EPA + DHA treatments. mRNA expression levels of interleukin (IL)-6 and tumor necrosis factor (TNF)-α were hardly detectable after either treatment (< 100 copies/10^5^ copies ATP5o). mRNA expression of IL-18 and of the transcription factor signal transducer and activator of transcription 3 (STAT3) was higher (≥ 5000 copies/10^5^ copies ATP5o) but still relatively low. EPA + DHA supplementation had no impact on any of these levels (changes between −15 and +26% vs. placebo, all *P* ≥ 0.73). Despite these low levels of expression and the lack of change with EPA + DHA supplementation, treatment-induced variations in mRNA STAT3 levels (EPA + DHA vs. placebo) were correlated with concurrent variations in mRNA expression of IL-6 (*r* = 0.55; *P* = 0.06), IL-18 (*r* = 0.64; *P* = 0.03), and TNF-α (*r* = 0.67; *P* = 0.02).

**Table 3:** Inflammatory gene expression in duodenal tissue after EPA + DHA and placebo supplementation in 12 men with type 2 diabetes

	**Placebo**	**EPA + DHA**	**Difference**	***P***
	**mRNA copies/10**^**5 **^**copies ATP5o**			
IL-6	8 (9)	10 (3)	2	0.77
TNF-α	109 (51)	93 (50)	−16	0.75
IL-18	5226 (3552)	5398 (1486)	172	0.73
STAT3	4637 (1723)	4646 (1437)	9	0.91

Data are expressed as median and interquartile range in parentheses. *P* values were calculated using Wilcoxon matched-pairs signed-rank tests. Abbreviations: *ATP5o*, ATP synthase O subunit; *DHA*, docosahexaenoic acid; *EPA*, eicosapentaenoic acid; *IL*, interleukin; *mRNA*, messenger ribonucleic acid; *STAT3*, signal transducer and activator of transcription 3; *TNF-α*, tumor necrosis factor α.

## Discussion

To the best of our knowledge, this study is the first to assess the impact of EPA + DHA supplementation on the expression of inflammatory genes in duodenal tissues of obese patients with type 2 diabetes.

Our results first showed that the expression of inflammatory genes in duodenal tissues of obese men with type 2 diabetes is relatively low, particularly in the case of IL-6 and TNF-α. This suggests that inflammation at the level of duodenal cells *per se* may not play a significant role in modulating chronic low-grade inflammation in obese diabetic patients. As reviewed by Ding and Lund [4], very little is known on the crosstalk between the intestine and adipose tissue in fostering chronic inflammation. In the case of Crohn’s disease, the extent of inflammation and cellular damage has been correlated with the accrual of mesenteric fat around the intestine (so-called “creeping” fat) [17]. As is the case with abdominal fat, mesenteric fat is characterized by infiltration with immune cells and increased levels of pro-inflammatory cytokines such as IL-6 and TNF-α [18]. The duodenal biopsy procedure in the present study collected basolateral cells and not cellular material from the outer surface of the intestine where mesenteric fat would be found. Thus, our study cannot resolve the possibility that mesenteric fat expresses pro-inflammatory genes in type 2 diabetes.

Our study also suggests that 3 g/d EPA + DHA supplementation has no impact on duodenal cells gene expression of pro-inflammatory cytokines and of transcription factor STAT3 compared with a placebo. Previous studies in animal models have shown that LCn-3PUFA from fish oil down-regulate the small intestine and gut-associated lymphoid tissue expression of inflammatory mediators (interferon-γ, chemokines) [19,20]. Other recent studies in cell culture and animal models also suggest that LCn-3PUFA down-regulate inflammatory gene expression by modifying the activity of transcription factors such as nuclear factor-κB and peroxisome proliferator-activated receptor-γ, which are expressed in the gastrointestinal tract [21]. The lack of effect of LCn-3PUFA supplementation on mRNA expression of pro-inflammatory cytokines and of transcription factor STAT3 in duodenal tissues of obese patients with type 2 diabetes in the present study is most likely attributable to the fact that gene expression was extremely low and therefore unlikely to be further modified. However, consistent with our results, a study performed in 242 human subjects by Pot et al. [22] has shown that concentrations of local markers of inflammation measured in biopsy samples of the colon were unaffected by consumption of oily fish, which represents a naturally occurring source of EPA + DHA.

A limitation that needs to be pointed out is the limited number of subjects which yielded limited statistical power to detect treatment differences in gene expression. However, mRNA expression levels of inflammatory genes were very low in most cases, and it is unlikely that such numbers would have changed with a larger sample size. The use of a randomized crossover design is strength and the long intervention and washout periods have limited the possibility of a carry-over effect of the LCn-3PUFA supplementation. Analyses of plasma fatty acid profiles following placebo and EPA + DHA supplementation confirmed compliance to treatments.

In summary, we believe this is the first study suggesting that gene expression of pro-inflammatory cytokines in duodenal tissues from obese patients with type 2 diabetes is very low and not affected by EPA + DHA supplementation. Further studies will be needed to investigate if inflammatory gene expression in other tissues surrounding the intestine such as mesenteric fat is modulated by EPA + DHA supplementation.

## Abbreviations

ATP5o: ATP synthase O subunit; BMI: Body mass index; DHA: Docosahexaenoic acid; DNA: Deoxyribonucleic acid; EPA: Eicosapentaenoic acid; HbA1c: Hemoglobin A1c; IL: Interleukin; LCn-3PUFA: Long-chain omega-3 polyunsaturated fatty acids; mRNA: Messenger RNA; RNA: Ribonucleic acid; STAT3: Signal transducer and activator of transcription 3; TG: Triglycerides; TNF-α: Tumor-necrosis factor α.

## Competing interests

The authors declare that they have no competing interests.

## Authors’ contributions

PC, VL and BL designed the research; AJT and JCH coordinated the study and participated in the data collection; VL performed duodenal biopsies; MEL and BL performed statistical analyses; MEL interpreted the data and wrote the manuscript; BL had primary responsibility for final content. All authors critically reviewed the manuscript and approved its final version.

## Supplementary Material

Additional file 1 **Details on RNA quantification and quantitative real-time PCR.** This file provides complete details on RNA quantification and quantitative real-time PCR performed in the present study and describes primer sets used in quantitative real-time PCR from duodenal biopsies. Click here for file
