# Supplementary material for: Eicosapentaenoic and docosahexaenoic acid supplementation and inflammatory gene expression in the duodenum of obese patients with type 2 diabetes
Source: Nutr J. 2013 Jul 15;12:98. doi: 10.1186/1475-2891-12-98 (PMC3718629; doi:10.1186/1475-2891-12-98)
Supplement: Additional file 1 — Details on RNA quantification and quantitative real-time PCR. This file provides complete details on RNA quantification and quantitative real-time PCR performed in the present study and describes primer sets used in quantitative real-time PCR from duodenal biopsies. [file 1475-2891-12-98-S1.pdf]

Additional file 1.

Details on RNA quantification and quantitative real-time PCR

RNA quality was assessed with an Agilent 2100 Bioanalyzer (Agilent Technologies Inc). First-strand complementary DNA (cDNA) synthesis was accomplished using 5 µg of isolated RNA in a reaction containing 200 U of Superscript III RNase H-RT (Invitrogen Life Technologies), 300 ng of oligo-dT_{18}, 50 ng of random hexamers, 50 mM Tris-HCl pH 8.3, 75 mM KCl, 3 mM $MgCl_2$, 500 µM deoxynucleotides triphosphate, 5 mM dithiothreitol, and 40 U of Protector RNase inhibitor (Roche Diagnostics); the volume of the final solution was 50 µl. The reaction was performed at 25°C for 10 minutes, followed by 50°C for 1 hour, and then the solution was treated with 1 µg of RNase A for 30 min at 37°C. The resulting products were purified with Qiaquick PCR purification kits (QIAGEN). cDNA corresponding to 20 ng of total RNA was used to perform fluorescent-based real-time PCR quantification using the LightCycler 480 (Roche). Reagent LightCycler 480 SYBRGreen I Master was obtained from the same company and used according to the manufacturer's instructions. Fifty cycles of PCR reactions were performed under the following conditions: denaturation at 95°C for 10 sec, annealing at 58-64°C for 10 sec and elongation at 72°C for 14 sec. The reaction was then heated for 5 sec at a temperature 2°C lower than the melting temperature of the DNA fragment. The fluorescence signal readings were recorded at the end of the heating period to avoid a non-specific signal. A melting curve was performed to assess for the presence of a non-specific signal. Oligoprimer pairs that amplify approximately 200 bp were designed using GeneTools software (Biotools Inc.), and their specificity was verified by blast in the GenBank database. Data analyses and normalization were performed using the second-derivative and double-correction methods as described by Luu-The *et al*. [1] (see references below); the following reference genes were used: hypoxanthine guanine phosphoribosyl transferase 1 (Hprt1), ATP synthase O subunit (ATP5o), glucose-6-phosphate dehydrogenase (G6PD) and 18S ribosomal RNA (18S). Previous studies have shown that Hprt1, ATP5o and G6PD have stable expression levels from the embryonic stage of life through adulthood in various tissues [1]. The mRNA expression levels are expressed as the number of copies/10^5 copies of the reference gene ATP5o, using a

standard curve of crossing points versus the logarithm of the quantity. The standard curve was established using known amounts of purified PCR products (10, 10^2, 10^3, 10^4, 10^5, and 10^6 copies) and the LightCycler 480 version 1.5 software provided by the manufacturer (Roche Inc.). The efficiency of the PCR amplification was verified.

Primer sets used in quantitative real-time PCR from duodenal biopsies

Gene Symbol	Description	Forward Primer (5'→3')	Reverse Primer (5'→3')
**IL-6**	Interleukin 6 (interferon, beta 2)	ACAGCCACTCACCTCTTCAGA	AGTGCCTCTTTGCTGCTTTCA
**TNF-α**	Homo sapiens tumor necrosis factor	CATCTATCTGGGAGGGGTCTT	GCAATGATCCCAAAGTAGACC
**IL-18**	Homo sapiens interleukin 18 (interferon-gamma-inducing factor)	GCTGAAGATGATGAAAACCTGGAAT	ATAAATATGGTCCGGGGTGCA
**STAT3**	Homo sapiens signal transducer and activator of transcription 3 (acute-phase response factor)	GGTTGGACATGATGCACACTAT	AGGGCAGACTCAAGTTTATCAG
**ATP5o**	ATP synthase, H+ transporting, mitochondrial F1 complex, O subunit	ATTGAAGGTCGCTATGCCACAG	AACGACTCCTTGGGTATTGCTTAA

References

[1] Luu-The V, Paquet N, Calvo E, Cumps J: **Improved real-time RT-PCR method for high-throughput measurements using second derivative calculation and double correction.** *Biotechniques* 2005, **38:**287-293
